# Cloning and expression analyses of a *Pyrabactin Resistance 1 (PYR1)* gene from *Magnolia sieboldii* K. Koch

**DOI:** 10.1080/21655979.2021.1947168

**Published:** 2021-07-05

**Authors:** Wan-Qi Zeng, Hong-Tao Sun, Lei Wang, Xiu-jun Lu, Xiao-lin Zhang

**Affiliations:** a Department of Forestry, Shenyang Agricultural University, Shenyang, China; b State Key Laboratory of Tree Genetics and Breeding, Research Institute of Forestry, Chinese Academy of Forestry, Beijing, China

**Keywords:** *Magnolia sieboldii* K. Koch, ABA signal, *MsPYR1*, prokaryotic expression, expression analysis

## Abstract

*Magnolia sieboldii* K. Koch is endemic to China and has high medicinal and ornamental values. However, its seed exhibits morphophysiological dormancy, and the molecular mechanisms of which are not clearly understood. To reveal the regulation mechanism of the ABA signal in seed dormancy, the *M. sieboldii* ABA receptor *Pyrabactin Resistance* 1 (*PYR1*) gene was cloned and analyzed. Analysis of the *MsPYR1* sequence analysis showed that the full-length cDNA contained a complete open reading frame of 987 bp and encoded a predicted protein of 204 amino acid residues. The protein had a relative molecular weight of 22.661 kDa and theoretical isoelectric point of 5.01. The transcript levels of *MsPYR1* were immediately upregulated at 16 DAI and then decreased at 40 DAI. The highest transcript level of *MsPYR1* was found in the dry seeds, indicating that the *MsPYR1* gene may play an important role in the regulation of dormancy. The *MsPYR1* gene cDNA was successfully expressed in *E. coli* Rosetta (DE3), and the protein bands were consistent with the prediction. The Anti-MsPYR1antibody could detect the expression of MsPYR1 in *M. sieboldii*. The results provided a foundation for further study of the function of the *MsPYR1* gene.

ABBREVIATIONS

ABA: Abscisic acid; MPD: morphophysiological; PYR1: Pyrabactin Resistance1; PYL: Pyr1-Like; RCAR: Regulatory Components of Aba Receptors; PP2C: protein phosphatases 2C; SnRK2: sucrose non-fermenting1-related protein kinase2; DAI: day after imbibition; NCBI: National Center for Biotechnology Information; BCA: Bicinchoninic acid; CDD: Conserved Domains.

## Graphical Abstract

**Figure:**
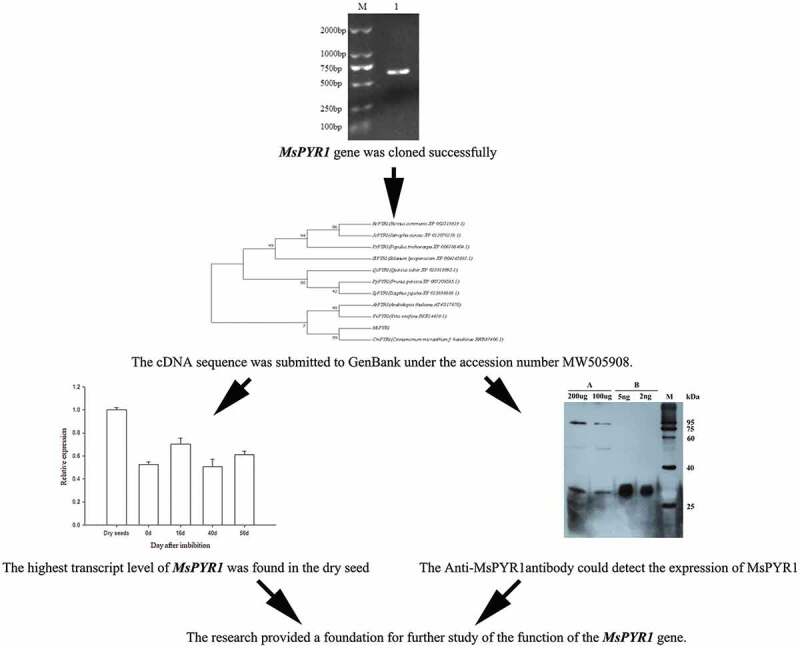

## Introduction

*Magnolia sieboldii* K. Koch is widely distributed in China, Korea, and Japan. *M. sieboldii* has high ornamental and medicinal values [1]. The plant had been designated as the City Flower of Benxi in P.R. China and the National Flower of North Korea. Given the difficulty of natural regeneration, the population of *M. sieboldii* has been decreasing. *M. sieboldii* has been classified in the IUCN Red List because of ‘least concern’ [2]. The seed dormancy type belongs to the levels of morphophysiological (MPD) [3]. Abscisic acid (ABA) can affect seed germination by modulating ABA-related functional genes. However, the molecular mechanism of the MPD of the *M. sieboldii* seed remains unknown.

The phytohormone ABA regulates plant growth and development processes, such as seed dormancy, fruit ripening, leaf senescence, and stress resistance [4]. ABA signaling is required for vegetative and reproductive growth, stomatal aperture, and transcriptional response to the hormone [5]. The ABA signal transduction cascade is one of the best characterized input transmission pathways of plants at the molecular level. The ABA signaling pathway has identified a large number of proteins [6]. Pyrabactin Resistance1 (PYR1)/Pyr1-Like (PYL)/ Regulatory Components of Aba Receptors (RCAR) ABA receptors, clade A type protein phosphatases 2 C (PP2C) and group III sucrose non-fermenting1-related protein kinase2 (SnRK2) subfamily are essential core components of the upstream signal transduction network that regulates ABA-responsive processes, including dormancy and germination [7–10].

The PYR/PYL/RCAR ABA receptor specifically perceives ABA. The PYR/PYL/RCAR ABA receptors have identified 15 members in the tomato [11] and 14 members in Arabidopsis [5]. PYR1 is critical for seed germination and seedling establishment [10], positive regulation of fruit ripening [12], reducing vegetative growth and seed production [5], inducing stomatal closing movement [13], and response to dehydration stress [14]. The expression of PYR1 in the seeds is higher than that in the other tissues [15].

PYR1 is necessary for ABA signaling in vivo. A study has shown that ABA binds to PYR1 and inhibits PP2Cs [15]. The primary objective of the present study was to clone the *PYR1* gene from *M. sieboldii*, study its expression at different germination stages. An attempt was made to express MsPYR1 protein. The cloning, transcription, and translation analysis of the *PYR1* gene provide a foundation for further study on the molecular mechanism of the ABA signal.

## Materials and methods

### Plant material and germination assays

*M. sieboldii* trees were grown at the Shenyang Botanical Garden (41° 850′ N, 123° 727′ E, 28 masl) in Shenyang, Liaoning, China. Fresh matured seeds were collected from 20 trees with average height of 7 m on 20 October 2018. The coat was removed, and the seeds were surface sterilized using potassium permanganate (0.05% [*w/w*]) for 15 min and rinsed thrice with sterilized distilled water. The seeds were allowed to imbibe different treatments, i.e., 10 mM ABA and water, in Petri dishes at 20°C for 48 h. Each treatment had three biological replicates of 100 seeds per Petri dish. After imbibition, the seeds were cold stratified in the dark at 4°C. Seed samples were collected at 0 h imbibition (dry seed, GZ), 0 day after imbibition (DAI) and every 8th DAI then stored at −80°C for RNA extraction.

### RNA extraction and cDNA synthesis

The five key time points for seed germination were as follows: dry seed, 0 DAI, 16 DAI, 40 DAI, and 56 DAI. The seeds were collected at these time points and used for total RNA extraction. The RNA was extracted using the RNA Simple Total RNA Kit (Tiangen, Beijing, China) [16]. The concentration and purity of the RNA obtained were detected using Nanodrop2000 UV electrophotometer (Thermo Scientific). The cDNA was synthesized using the GoScript^TM^ Reverse Transcription System (Promega).

### PYR1 *gene cloning and sequencing*

The full length of the primers of *PYR1* (Table 1) was designed based on the transcriptome data by using the Primer 5 software (unpublished). The *PYR1* clone was amplified using PrimeSTAR® HS DNA Polymerase (TaKaRa). RT-PCR reaction system was used in a 50 μL of reaction mixture including PrimeSTAR HS Taq (25 μL), primers (2 μL), cDNA template (1 μL), and ddH_2_O. The RT-PCR reaction conditions were as follows: 30 s at 98°C; 30 cycles of 10 s at 98°C, 15 s at 58°C, 1 min at 72°C; and a 10 min extension at 72°C. The PCR fragments were connected into the pMD18-T vector (TaKaRa) and sequenced by TaKaRa Co., Ltd.

**Table 1.** Primers used in this research

Primer names	Sequence
*MsPYR1*-F	5ʹ-ATGGAAGAAGGAGAGAAATCAAC-3’
*MsPYR1*-R	5ʹ-TTATCCTCCCTTTTGATCCTC-3’
*MsPYR1*-qF	5ʹ-GCAATACAGGTCGGTGACGA-3’
*MsPYR1*-qR	5ʹ-ATCCTCCTTCGCCATACCCT-3’
*Ms60S rRNA*-qF	5ʹ-TTGATTCGGTCTATGGTTCGTT-3’
*Ms60S rRNA*-qR	5ʹ-GTTCAGATTCTTGAGCGGGTT-3’
*pET28a-F*	5ʹ-TAATACGACTCACTATAGGG-3’
*pET28a-R*	5ʹ-GCTAGTTATTGCTCAGCGG-3’

### Quantitative real-time PCR (qRT-PCR)

The qRT-PCR procedure was established using the qTOWER 2.0/2.2 Real Time PCR Systems (USA). All primers are listed in Table 1. The qRT-PCR reaction system was used in a 20 μL reaction mixture including PrimeSTAR HS Taq (10 μL), primers (1.6 μL), cDNA template (2 μL), and ddH_2_O (6.4 μL). The qRT-PCR reaction conditions were as follows: 30 s at 95°C; and 40 cycles of 5 s at 95°C, 20 s at 60°C. The data were analyzed using the 2^−ΔΔ^Ct method described by Zhang et al [17].

### Bioinformatics analysis

The open reading frame (ORF) and sequence alignment were analyzed using DNAMAN version 6.0. The transmembrane regions were predicted using TMHMM 2.0 (http://www.cbs.dtu.dk/services/TMHMM/). The structural regions were predicted using the National Center for Biotechnology Information (NCBI) (https://www.ncbi.nlm.nih.gov/Structure/cdd/wrpsb.cgi). The protein motif was conducted by using the Simple Modular Architecture Research Tool (SMART) (http://smart.embl-heidelberg.de/). The phylogenetic tree was constructed using Clustalx2.1 and MEGA7.0 [18].

### *Prokaryotic expression of the* PYR1 *gene*

The prokaryotic expression vector pET28a-PYR1 was constructed by PCR with pMD18-T-PYR1 as template. The pET28a-PYR1 construct was transformed into *E. coli* Rosetta (DE3). The positive clones were screened by PCR with pET28a-F and pET28a-R as primers (Table 1). The monoclones were selected and inoculated in fresh LB liquid medium (containing 50 mg/L kanamycin) for approximately 4 h at 37°C, and the OD_600_ was approximately 0.6. IPTG with a final concentration of 0.5 mM was added to induce the expression of PYR1 [19].

### Purification of the recombinant protein

The bacterial culture solution was induced at 15°C for 15 h, and then the lysed cell was ultrasonicated. The cell lysates were loaded to the chromatographic columns with Ni IDA resin. The column was washed with 200 mL of the wash buffer containing 2 M NaCl and 15 mL of the wash buffer containing 50 mM NaCl. The proteins were eluted with 20 mL of an elute buffer containing 20 mM iminazole, then the proteins were eluted with 20 mL of another elute buffer containing 250 mM iminazole [20]. Approximately 1 mg/mL of pure recombinant PYR1 was obtained.

### Antibody preparation and western blot analyses

After the concentration of the Bicinchoninic acid (BCA) protein was determined, two New Zealand rabbits (2–2.5 kg) were injected subcutaneously with 400 µg, immunized once at 1.5 weeks and the process was performed four times. Blood samples were collected for detection, and the titer of the antiserum against PYR1 was determined by indirect ELISA. The titer was greater than 1:500,000, and the final blood samples were collected to prepare the antiserum. Western blot analysis was performed as described [21]. After 12% SDS-PAGE electrophoresis, the gels were transferred onto a Trans-BlotTurboTM (BioRad) transfer system. Anti-PYR1 antibody (1:500) was used as the primary antibody and anti-rabbit IgG (1:5000) as the secondary antibody.

## Results and discussion

### *Cloning and sequence analysis of* MsPYR1

**Figure 1.**
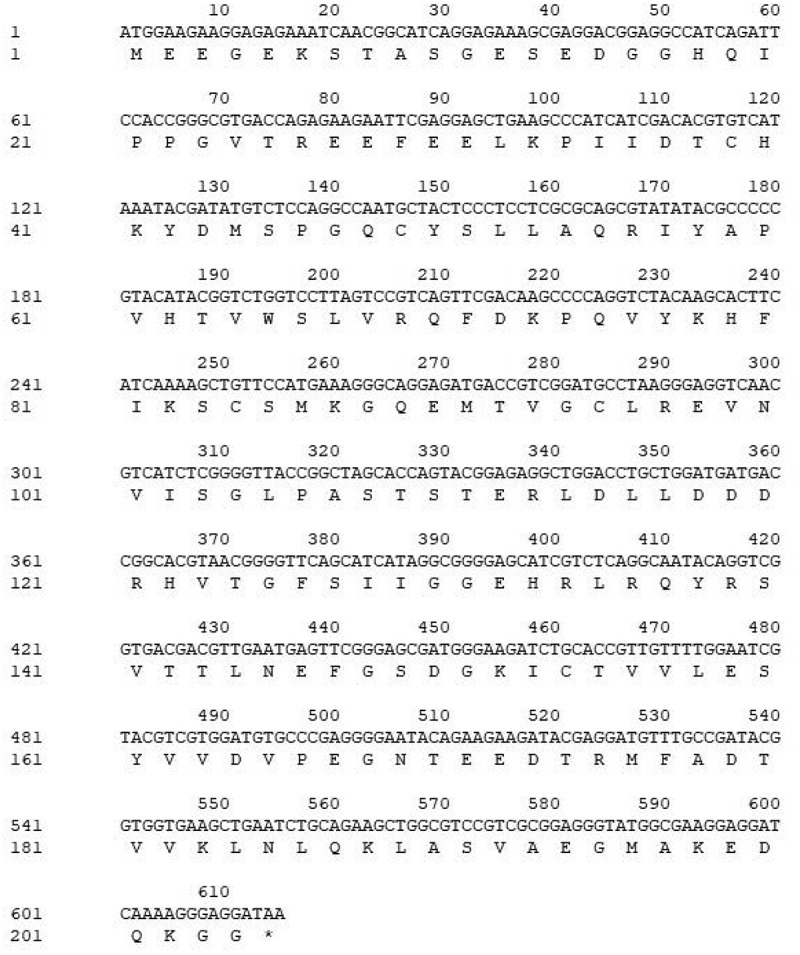
The cDNA of *MsPYR1* and its encoded amino acid sequence

The full-length *PYR1* cDNA was obtained from *M. sieboldii* seed by RT-PCR. The length of the PCR product contained a complete ORF of 615 bp and encoded a predicted protein of 204 amino acid residues (Figure 1). The cDNA sequence was submitted to GenBank under the accession number MW505908.

**Figure 2.**
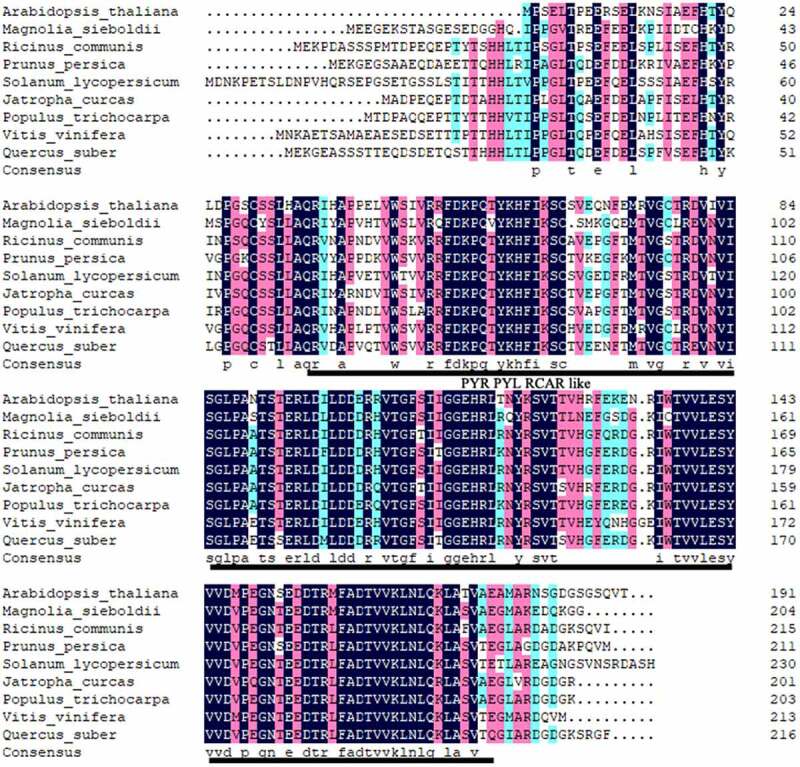
Comparison of the predicted protein sequence of BrcSPL8 with other SPL8 proteins

Multiple amino-acid sequence alignment revealed homology between the predicted amino-acid sequence of MsPYR1 and other PYR1 proteins (Figure 2). MsPYR1 showed an amino-acid sequence similarity of 65.22% with PtPYR1 from *Populus trichocarpa*, 64.65% with VvPYR1 from *Vitis vinifera*, 64.45% with PpPYR1 from *Prunus persica*, 61.95% with JcPYR1 from *Jatropha curcas*, 61.86% with RcPYR1 from *Ricinus communis*, 61.47% with QsPYR1 from *Quercus suber*, 61.04% with SlPYR1 from *Solanum lycopersicum* and 57.62% with AtPYR1 from *Arabidopsis thaliana*.

**Figure 3.**
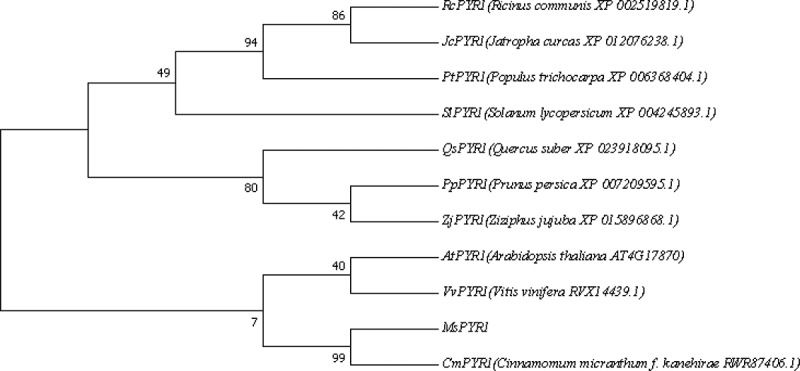
Sequence alignment of MsPYR1 and PYR1 from different plants

To evaluate the molecular evolutionary relationships among MsPYR1 and other PYR1s, the phylogenic tree was constructed using the amino acid sequence through the MEGA7.0 software. MsPYR1 was more closely related to *Arabidopsis thaliana, Vitis vinifera*, and especially *Cinnamomum micranthum* (Figure 3).

### Bioinformatic analysis of MsPYR1

The relative molecular weight of the MsPYR1 protein was 22.661 kDa, and the theoretical isoelectric point of the protein was 5.01. The amino acid with the highest content in the protein was Glu (9.8%), and the lowest content was Trp (0.5%) (Table 2).

**Table 2.** The amino acid composition of *MsPYR1* gene encodes the protein

amino acid residue	ratio	amino acid residue	ratio
Ala (A)	3.9%	Leu (L)	7.4%
Arg (R)	4.9%	Lys (K)	5.9%
Asn (N)	2.0%	Met (M)	2.9%
Asp (D)	6.4%	Phe (F)	2.9%
Cys (C)	2.5%	Pro (P)	3.9%
Gln (Q)	4.4%	Ser (S)	7.8%
Glu (E)	9.8%	Thr (T)	6.9%
Gly (G)	8.8%	Trp (W)	0.5%
His (H)	2.9%	Tyr (Y)	2.9%
Ile (I)	4.4%	Val (V)	8.8%

**Figure 4.**
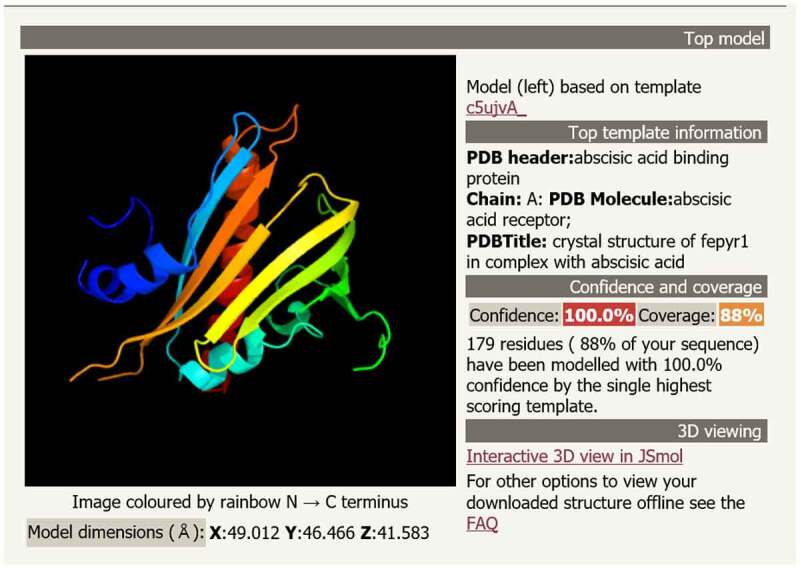
Prediction of three-dimensional structure of MsPYR1 protein

The conserved domain of the MsPYR1 protein was predicted by Conserved Domains (CDD) in the NCBI. The MsPYR1 protein had a structural domain of the PYR/PYL/RACR protein family, which in turn belonged to the START/RHO_alpha_C/PITP/Bet_v1/CoxG/CalC superfamily and contained putative hydrophobic ligand binding sites, protein interfaces, and gate structures (Figure 2). The SMART analysis showed a Polyketide_cyc2 domain Leu^53th^ to Glu^194th^ (Figure 1). The three-dimensional structure of the MsPYR1 protein was predicted using Normal model and the crystal structure of FePYR1 was selected as a template, the sequence identity between MsPYR1 protein and FePYR1 protein was 88% (Figure 4).

### *Transcription analysis of* MsPYR1 *in the different stratification stages*

**Figure 5.**
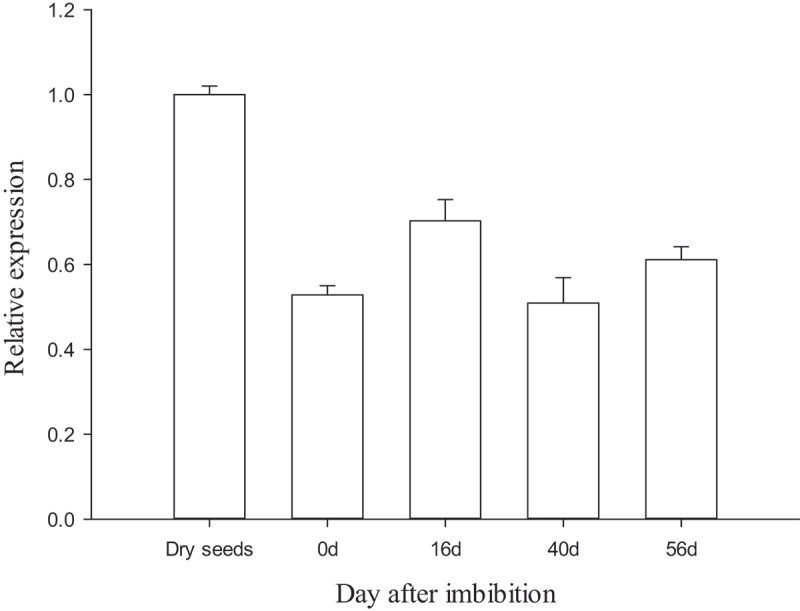
Expression of *MsPYR1* gene in different stratification stages

qRT-PCR was used to analyze the transcription of *MsPYR1* at different stratification stages (Figure 5). The highest *MsPYR1* transcript was detected in the dry seeds. During stratification, the PYR1 transcript was immediately upregulated at 16 DAI and then decreased. The lowest expression was observed at 40 DAI, during which the seed coat began to crack. Therefore, *MsPYR1* may play a vital role in the imbibition and seed germination. In *Arabidopsis, PYR/PYLs* were also expressed at high levels in the dry seeds, which were more sensitive to ABA [15]. PYR/PYLs play a crucial role in the regulation of seed dormancy and stomatal switch. Compared with the wild type, the *pyr/pyls* mutants were less sensitive to ABA [22,23]. This characteristic could explain the beginning of the cracking of the seed coat and the low expression level of *MsPYR1* at 40 DAI. So, ABA is a major plant hormone involved seed germination and early seedling development [24,25].

### Prokaryotic expression of MsPYR1 and immunoblot analysis

**Figure 6.**
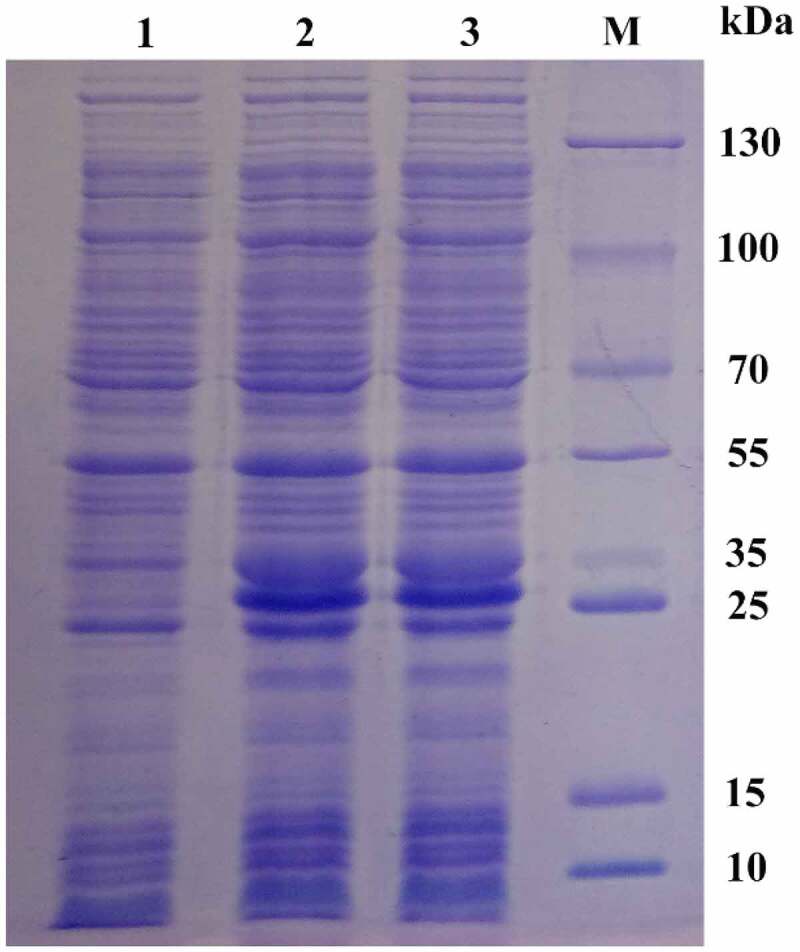
Analysis of the expression of recombinant pPID1 by SDS-PAGE Lanes 1: 0 mM IPTG induction. Lanes 2,3: 0.5 mM IPTG induction. Lan M: protein molecular weight marker

The recombinant plasmid PET28A-MSPYR1 was transformed into the *E. coli* Rosetta (DE3) cell. After induction with 0.5 mM IPTG at 37°C for 2 h, 12% SDS-PAGE electrophoresis was performed (Figure 6). The results showed that after IPTG induction, a protein band appeared near 26 kDa, which was slightly larger than the theoretical molecular weight of 22.661 kDa. This characteristic may be affected by the his-tag. After expansion, the MsPYR1 protein was purified by using 6× His protein purification kit. *E. coli* Rosetta (DE3) is frequently used for the high expression of eukaryotic or prokaryotic proteins [26,27].

**Figure 7.**
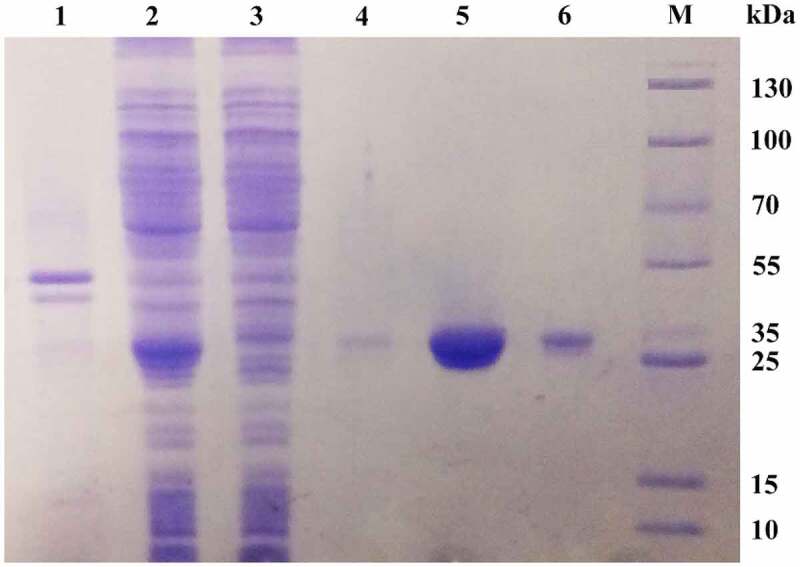
SDS–PAGE of purified recombinant protein MsPYR1 Lane 1: the precipitate of the crude cell lysate. Lan 2: the supernatant of the crude cell lysate. Lan 3: effluent fractions of loading sample. Lan 4: the fraction of the first elution with 20 mM imidazole eluate fractions. Lan 5: the fraction of the first elution with 250 mM imidazole eluate fractions. Lan 6: residual fraction. Lan M: protein molecular weight marker

The protein was loaded into a Ni-IDA affinity column to bind the polyhistidine-tagged recombinant MsPYR1. The purity of MsPYR1 eluted from the first Ni-IDA affinity column with 20 mM imidazole was approximately 20%. After purification of the second Ni-IDA affinity column with 250 mM imidazole, MsPYR1 was approximately 80%, which was exhibited as a single band on the SDS–PAGE with the molecular weight of 26 kDa (Figure 6). The expression product easily formed inclusion bodies, which increased the difficulty for protein purification [28]. To avoid the formation of inclusions and enhance the soluble yield of the target protein, the expression was performed at a low temperature (15°C) [29,30]. In this study, the *E. coli* Rosetta (DE3) strain had a low expression level of the recombinant proteins as inclusion bodies (Figure 7).

**Figure 8.**
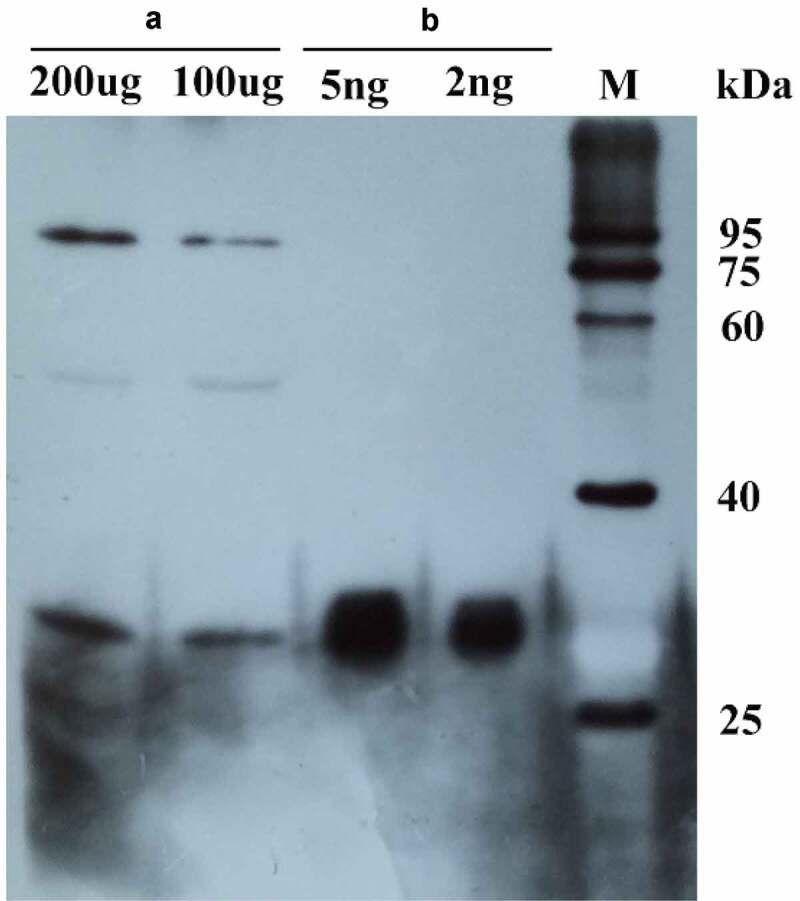
The purified MsPYR1 protein and seed protein was detected by western blotting analysis. A: *M. sieboldii* seed protein. B: MSPYR1 recombinant protein. M: protein molecular weight marker

Anti-MsPYR1 antibodies are used as a convenient detection tool to bind/recognize the target proteins with higher stability and affinity. The presence of a cross-reacting protein appeared at the approximate size of 26 kDa in the purification of recombinant proteins and seed protein during the immunoblot analysis (Figure 8). When the primary antibody was diluted 500 times, the recombinant protein (2 ng) and seed protein 100 (µg) could be detected by the assay.

## Conclusions

In the present study, an ABA receptor gene, *MsPYR1* was cloned and successfully expressed in *E. coli*. The expression pattern of *MsPYR1* at different stratification stages was analyzed using qPCR. The highest transcript level of *MsPYR1* was found in the dry seeds. The pure recombinant MsPYR1 protein was successfully obtained. The Anti-MsPYR1 antibody could detect the expression of *MsPYR1* in *M. sieboldii*. The results provided a foundation for further study of the function of the *MsPYR1* gene. The results of present study provide valuable information for further functional studies of MsPYR1 gene.
